# Microwave-Assisted Synthesis of Stretchable and Transparent Poly(Ethyleneglycol-Sebacate) Elastomers with Autonomous Self-Healing and Capacitive Properties

**DOI:** 10.1089/soro.2019.0148

**Published:** 2021-06-16

**Authors:** Gülçin Günal, Meltem Okan, Dincer Gokcen, Tuncer Caykara, Halil Murat Aydin

**Affiliations:** ^1^ Bioengineering Division, Institute of Science, Hacettepe University, Ankara, Turkey.; ^2^ Department of Micro and Nanotechnology, Graduate School of Natural and Applied Sciences, Middle East Technical University, Ankara, Turkey.; ^3^ Department of Electrical and Electronics Engineering, Faculty of Engineering, Hacettepe University, Ankara, Turkey.; ^4^ Department of Chemistry, Faculty of Science, Gazi University, Ankara, Turkey.; ^5^ Centre for Bioengineering, Hacettepe University, Ankara, Turkey.

**Keywords:** self-healing elastomer, artificial skin, soft robotics, capacitive sensor, microwave synthesis

## Abstract

Introducing functional synthetic biomaterials to the literature became quite essential in biomedical technologies. For the growth of novel biomedical engineering approaches, progressive functional properties as well as the robustness of the manufacturing processes are essential. By using acid-induced epoxide ring-opening polymerizations through catalysts, a wide variety of biodegradable and functionalized biomaterials can be synthesized. Sebacic acid (SA) and poly(ethylene glycol) diglycidyl ether (PEGDGE) are amongst the FDA-approved biocompatible materials. In this study, we focused on the rapid synthesis of caffeine-catalyzed self-healable elastomer via a facile microwave-assisted synthesis route. The elastomer prepared can be used in various applications, including tactile sensors, wearable electronics, and soft robotics. SA and PEGDGE were catalyzed in the presence of caffeine under microwave irradiation followed by crosslinking *in vacuo*, yielding an elastomeric material. The chemical characterizations of the obtained elastomer were carried out. The resulting material is transparent, highly stretchable, and has capacitive and self-healing properties even at room temperature. The material developed can be easily applied for the aforementioned applications.

## Introduction

Studies based on soft robotic systems made up of soft materials that can mimic the basic working principles of human body are accelerated in conjunction with the efforts made in the field. These soft robotic applications include the development of arms, soft grippers, robotic skins, and various wearable materials.^1^ With the recent developments in the field, the emphasis was placed especially on the development of soft materials, from which artificial skins can be produced that can imitate the native human skin structure and encode tactile stimuli. Compared with traditional robotic systems, the use of these kinds of soft materials provides the advantage of producing rather complicated structures with higher complexity and morphologies compatible with the environment. The main difficulty in their usage, however, with the infinite deformation feature of tactile sensors, is that the sense of touch is lost.^2^ At this point a new field emerged, where wearable sensors, actuators, antennas, and so on can be developed with skin-like electronics.^3^ The aim is to create artificial skins that can detect the tactile stimulus from their large surface areas. This kind of requirement could be met by soft sensors and actuators provided by elastomer-based flexible and stretchable electronics.^4–7^ The self-healing characteristics in the abovementioned synthetic polymers are provided through encapsulation of healing agents and inclusion of dynamic bonds.^8–12^

**FIG. 1.**
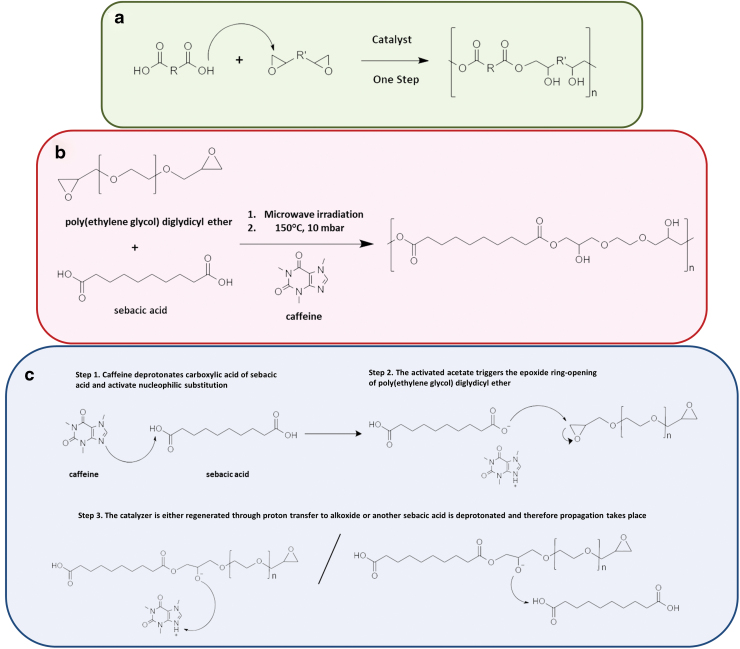
**(a)** General reaction mechanism between a di-epoxide and di-acid, **(b)** reaction mechanism for the synthesis of PEGSA via caffeine-catalyzed crosslinking of PEGDGE with SA, and **(c)** the detailed reaction mechanism for the production of PEGSA. PEGSA, poly(ethyleneglycol-sebacate); SA, sebacic acid. Color images are available online.

By using acid-induced epoxide ring-opening polymerizations using catalysts, a wide variety of biodegradable and functionalized biomaterials can be synthesized (Fig. 1a). These polymerization processes proceed in the presence of various functional groups, including aromatic, alkenyl, ester, ether, and hydroxyl. However, the reactions usually require high temperatures and long processing times.^13^ Recently, an active ingredient, caffeine, was used as a catalyst in the synthesis process of self-healable polymers and showed no toxic effect in crosslinked thermoset applications compared with traditionally used catalysts.^14^ The multifunctional carboxylic acids and monomers containing epoxides allow the production of adaptable network properties. Materials developed from this concept are reported to be easily carried forward for the clinical applications.^14^ DiCiccio *et al.* synthesized crosslinked gels using caffeine as a catalyst based on basic polyester chemistry and showed that they could be healed at 90°C in a period of 12 h.^14^ Moreover, the obtained gels showed an elongation at break value of 173%. In this study, unlike the synthesis route used by DiCiccio *et al.*, an elastomer was synthesized using an FDA-approved diacid monomer, namely sebacic acid (SA) instead of a triacid. The SA was attached to diglycidyl ether-modified poly(ethylene glycol) (PEGDGE) under microwave irradiation and in the second step of the process, the structure was then crosslinked *in vacuo* at 150°C. The resulting elastomer was found to be very flexible, provided enhanced elongation properties, and showed self-healing capability at room temperature. Surprisingly, this material was also found to have a capacitive response, which makes it a potential biodegradable elastomer to be used in the aforementioned applications.

The chemical characterization of the obtained elastomer was carried out and its self-healing property was mechanically evaluated without the need of any heat treatment. Finally, its capacitive characteristics were determined. Although the synthesis of covalently crosslinked thermosets might be considered challenging, using caffeine as a catalyst and employing microwave irradiation and vacuum as the triggering mechanisms, we were able to accomplish a partially crosslinked elastomer through a reaction between SA and PEGDGE.

## Materials and Methods

The facile synthesis route can be summarized as follows. To synthesize caffeine-catalyzed poly(ethyleneglycol-sebacate) (PEGSA), equal molar amounts (6 × 10^−3^ mol) of SA (Sigma, USA), caffeine (Sigma), and PEGDGE (Sigma) were mixed and placed in a glass Petri dish. The mixture was transferred to a microwave oven (Vestel, Turkey) to allow polymerization to take place. This procedure was adapted from Aydin *et al*.^15^ The microwave irradiation (595 W) was applied for 3 min (30 s × 6) with 15-s intervals. Once the prepolymerization step was completed, Petri dishes were transferred to a vacuum oven (Memmert, Germany) and prepolymers were cured at 150°C under vacuum (10 mbar) for 24, 32, 40, and 48 h.

The chemical composition of the PEGSA elastomer was characterized by attenuated total reflectance/Fourier transform infrared spectroscopy (ATR-FTIR) (Agilent, USA). Samples crosslinked for 24, 32, 40, and 48 h were directly placed on the ZnSe ATR crystal and measured in the range of 650–4000 cm^−1^. ^1^H NMR analysis (Bruker, USA) was used to determine the soluble extracts from the elastomer. For this aim, 20 mg of the extract was placed in the NMR tube and dissolved in 1 mL of dimethyl sulfoxide (Sigma) before the analysis. Tetramethylsilane (TMS) was used as a reference material.

The dynamic contact angles of the PEGSA elastomers were measured by a goniometer (Biolin Scientific, Sweden-Finland), using distilled/deionized water (4 μL, 18 MΩ cm resistivity) at room temperature. For each sample, at least five measurements from different surface locations were averaged.

The tensile test was performed to determine the mechanical characteristics, for which PEGSA materials with dimensions of 1 cm × 1 cm × 2 mm (*H* × *W* × *T*) were used (CellScale, Canada) and 4 samples from each group were tested at room temperature using a 50 N load cell at a rate of 5 mm/min. Young's moduli were calculated based on the stress/strain curve in the range between 20% and 40% strain. A cyclic tensile test was also performed on each elastomer with the same dimensions using full loading for 120 s at a strain rate of 5 mm/s. Totally, three cycles were run for each sample.

To evaluate the viscoelastic properties of PEGSA elastomers, a rheometer (Malvern Kinexus, United Kingdom) was used in parallel plate configuration. Each sample (8 mm diameter) was loaded on the plate and tests were performed at room temperature. The elastic modulus (*G*′) and the viscous modulus (*G*′′) values were plotted between the frequency ranges of 0.01–100 Hz. The readings are performed at 5 Hz.

To investigate the surface morphologies, cut sections were coated with gold-palladium and imaged with scanning electron microscopy (SEM) (Carl Zeiss Evo 50, Germany).

To show the difference in the transparency between 0% and 100% strained groups, light transmittance values were recorded over the visible light spectrum using a microplate spectrophotometer (Epoch-BioTek, USA). Pristine and 100% stretched PEGSA elastomers (cured for 32 h) were cut into disks and transferred to a 12-well plate. The average of three measurements was read and the absorbance values of every 10 nm wavelength were plotted for each sample. Transmittance values were calculated according to Equation (1).

(1) $$T\left(\%\right)=10^{\left(2-A\right)},$$

where *A* is the absorbance and *T* is the transmittance value.

Dielectric constant and conductivity were also investigated for the PEGSA elastomers. The dielectric constant is a parameter that determines the capacitance of the material under the electric field. Equation (2) below gives the capacitance of a medium between two electrodes (probes).

(2) $$C=\varepsilon_{0}\varepsilon_{r}\frac{A}{d},$$

Here $\varepsilon_{r}$ is the dielectric constant (also known as relative permittivity), $\varepsilon_{0}$ is the permittivity of free space ($8.85\times 10^{-12}F∕m$), *A* is the area of the material in contact with the measurement probes, and *d* refers to the thickness of the material (medium). When the area and thickness are maintained constant, the dielectric constant stands as the fundamental parameter that determines the capacitance.

Conductivity refers to the ability of current flow through the material and it is inversely proportional to the resistivity. The conductivity measurement is crucial to investigate the electrical properties since it has a significant influence on the applications at which the synthesized materials can be used. The conductivity (σ) is found by Equation (3) below.

(3) $$\sigma =\frac{1}{\rho },where\rho =\frac{VA}{Il}.$$

ρ is the resistivity, *V* is the measured voltage, and *I* is the current flowing through the synthesized material. If the area (*A*) in contact with the probe and length (*l*) of the material are known, the 4-probe measurement technique can be used to measure the resistivity and thus the conductivity.

## Results and Discussion

In the proposed reaction, caffeine catalyzes anhydrous carboxylate ring-opening of ethylene oxide from diglycidyl ether with bi-functional crosslinking agent SA. By combining this approach with simple microwave polymerization step a novel elastomer synthesis path was therefore introduced (Fig. 1b). Highly reactive epoxy compounds have been extensively used as starting materials for the chemical modification of functional groups and polymers with pendant epoxide rings considered reactive polymers and have been used as starting materials to produce functional polymers. Also, it is well established by now that reactions between epoxy compounds and carboxylic acids proceed quite readily. In such a case product contains hydroxyl groups, which can be used as the reactive section for further chemical modification.^16^ The polymerization, here, begins first with the activation of carboxylate through deprotonation of SA by caffeine, creating a nucleophile that attacks to the ethylene oxide groups of PEGDGE and opens the ring to create ester bond between (Fig. 1c). The as-explained acid-induced epoxide ring-opening polymerization can be applied to various types of molecules. The major adversity of such a process is the side reaction that can occur between the epoxide group of the di-epoxide molecule and the hydroxyl groups of the product. Such a side reaction can result in undesirable crosslinking. The catalyst, caffeine, was used to eliminate such a situation by catalyzing the poly-addition reaction between the carboxylic acid of SA and the cyclic ether, which is the ethylene oxide group, of PEGDGE.^13^ Functional groups in the resulting prepolymer are the hydroxyl groups, for which the modification methods are well known.

**FIG. 2.**
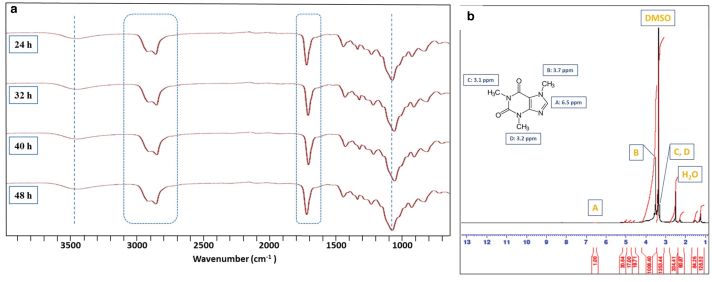
Fourier transform infrared spectra of the PEGSA elastomers crosslinked for 24, 32, 40 and 48 h, **(b)** ^1^H NMR spectrum of the soluble extracts of PEGSA elastomer (cured for 32 h). Color images are available online.

FTIR spectra of PEGSA elastomers crosslinked for 24, 32, 40, and 48 h are illustrated in Figure 2a. The peaks at 3447 cm^−1^ represent the free hydroxyl (OH) groups created upon the ring-opening of the epoxides of PEGDGE through SA attack. The peaks at 2922 and 2862 cm^−1^ are specific to C-H bonds. The esterification reaction between SA and PEGDGE was proven with the C = O peaks at 1729 cm^−1^. Finally, the peaks between the ranges of 1200–1000 cm^−1^ represent the C–O stretching. The hydroxyl peaks at 3447 cm^−1^ not affected by the curing time were also proven by the FTIR analysis. This showed that the elastomer structure reached the crosslinking saturation maxima after 24 h. The peaks at 1220 cm^−1^ are assigned to the combined stretching modes of C = O and –C–N bonds of caffeine, while the peak at 1596 cm^−1^ is attributed to C = N stretching vibration. The C–H bonds of methyl groups are overlapped with the other moieties.^17^ ^1^H NMR spectrum (Bruker) of the soluble extracts from the elastomer is given in Figure 2b. Only specific peaks of caffeine molecule were detected in the spectrum. This confirmed that monomers were completely incorporated into the structure. The peaks corresponding to the caffeine hydrogens appeared at ∼6.5 and 3.0–3.7 ppm. The FTIR and ^1^H NMR data together proved that the esterification reaction was successfully completed. The peak assignments at 1.2 and 1.5 ppm were attributed to small amounts of extracted unreacted SA.

**FIG. 3.**
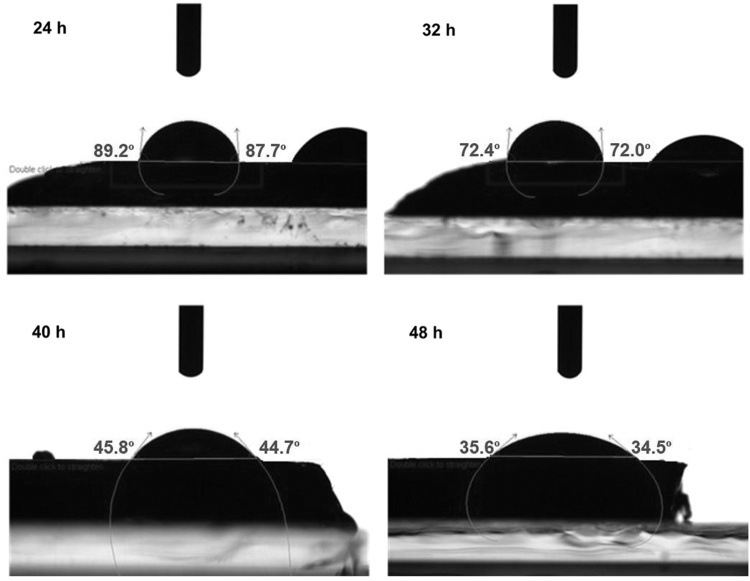
Measurements of advancing and receding angles of PEGSA elastomers at different curing times.

**FIG. 4.**
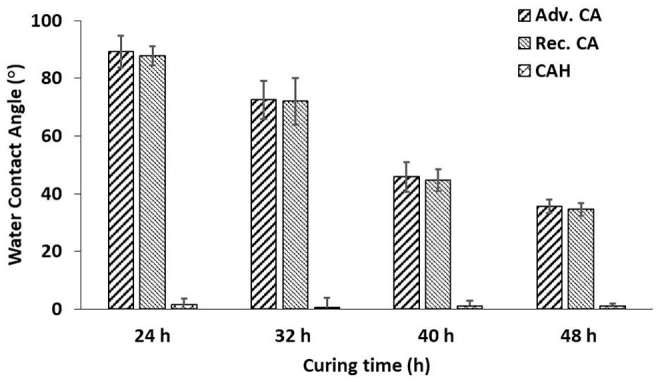
Variation of advancing (Adv.CA) and receding (Rec.CA) water CAH values with curing time. CAH, contact angles and hysteresis.

Hydrophilicity and surface characteristics of PEGSA elastomers were determined via the dynamic contact angle measurements. Exemplary profiles of the droplets were visualized by a camera and given in Figure 3. Their advancing and receding angles (*θ*_A_/*θ*_R_) are also given in Figure 4. As shown in Figure 4, *θ*_A_/*θ*_R_ values decreased from 89.2°/87.7° to 35.6°/34.5° as curing time increased from 24 to 48 h, indicating the increase of the hydrophilicity of PEGSA. This may be due to the surface enrichment of the free chains containing etheric groups with high chain flexibility. Similarly, this phenomenon is also observed in polymer-liquid surface studies, which is related with the reversible rearrangement of the polymer.^18,19^ The surface adhesion increased with contact period is directly dependent upon the molecular mobility of the surface, viscosity, and stiffness. Meanwhile, the partial reduction of the hysteresis values with increasing curing time shows that surface roughness reduced (Fig. 4). The increase of the crosslinking density with increasing curing time led to the formation of the smoother surface.

The values related to mechanical features of the polymer, such as tensile strength (kPa), elongation (%), and Young's modulus (MPa), depending on the curing time are provided in Table 1. The hydroxyl (–OH) groups exposed from the epoxide rings helped creating a crosslinked network between the chains *in vacuo*, and the increase in curing time led to an improvement in the tensile strength and Young's modulus values; however, the increased curing time induced a decrease in the elongation percentage in the material as expected due to increased restriction effects of the crosslinked joints on chain mobility. Therefore, PEGSA elastomer cured for 32 h was found to be optimal in terms of both elongation and handling features. In this study, SA polymer chains led to an incremental stretching property and resulted in 700% elongation while, in a similar study, maximum elongation percentage of the gels catalyzed with caffeine was determined as 173%.^14^ This remarkable stretching characteristic can be attributed to the selection of longer chain monomer (SA) with 10 carbon atom and different crosslinking sites utilized in the second stage of the proposed process.

**Table 1.** Mechanical Properties of the Prepared Poly(Ethyleneglycol-sebacate) Elastomers

Curing time (h)	Tensile strength (MPa)	Elongation (%)	Young's modulus (MPa)
24	0.09 (± 0.01)	644.7 (± 67.8)	0.0178 (± 0.003)
32	0.11 (± 0.04)	702.3 (± 72.3)	0.0185 (± 0.002)
40	0.14 (± 0.02)	602.0 (± 31.5)	0.0239 (± 0.005)
48	0.16 (± 0.04)	427.3 (± 11.0)	0.0335 (± 0.006)

**FIG. 5.**
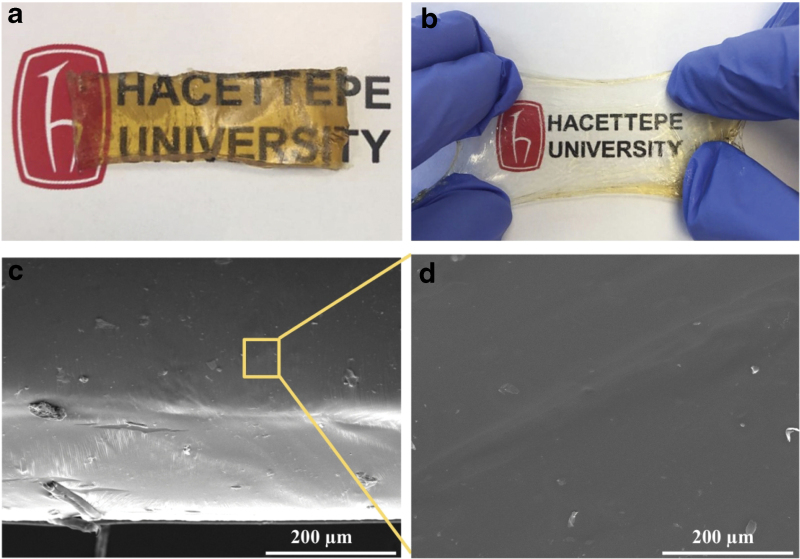
**(a)** Before and **(b)** after stretching images and **(c-d)** SEM images of PEGSA elastomer cured at 32 h (surface properties). Color images are available online.

Despite the successful results obtained from mechanical properties of PEGSA elastomers cured for 24 and 32 h, due to difficulties in handling and for being rather sticky, elastomer cured for 24 h was not found eligible to be used as a soft material. The crosslinked structures in the PEGSA elastomers were formed with the increase of curing time, and this led to deterioration in the self-healing and stretching features possibly due to consumption of the hydrogen bonds (Table 1). Therefore, PEGSA elastomer cured for 32 h was selected as the most suitable one to be used as a soft material. The structural analysis in both macro- and microlevels of this ideal elastomer is provided in Figure 5. For this, a section (3 × 1 cm) was cut from the elastomer and its stretching capability was shown morphologically.

**FIG. 6.**
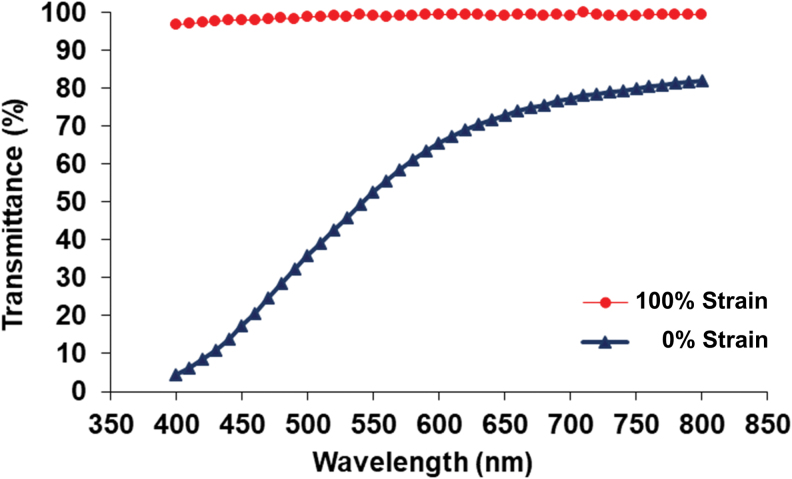
The transmittance values of PEGSA elastomers (cured for 32 h) recorded at 0% and 100% strain. Color images are available online.

It was found that the transparency values of the prepared elastomers were highly affected from the applied strains (Fig. 6). The light transmittance of 32 h-cured PEGSA elastomer was found as 52.58% ± 1.52% at 550 nm, while it was 98.20% ± 1.48% when the sample was stretched up to 100%. In addition to the changes observed in the capacitance with strain, this property was also thought to have a potential in the efforts toward construction of optical strain sensors.

**FIG. 7.**
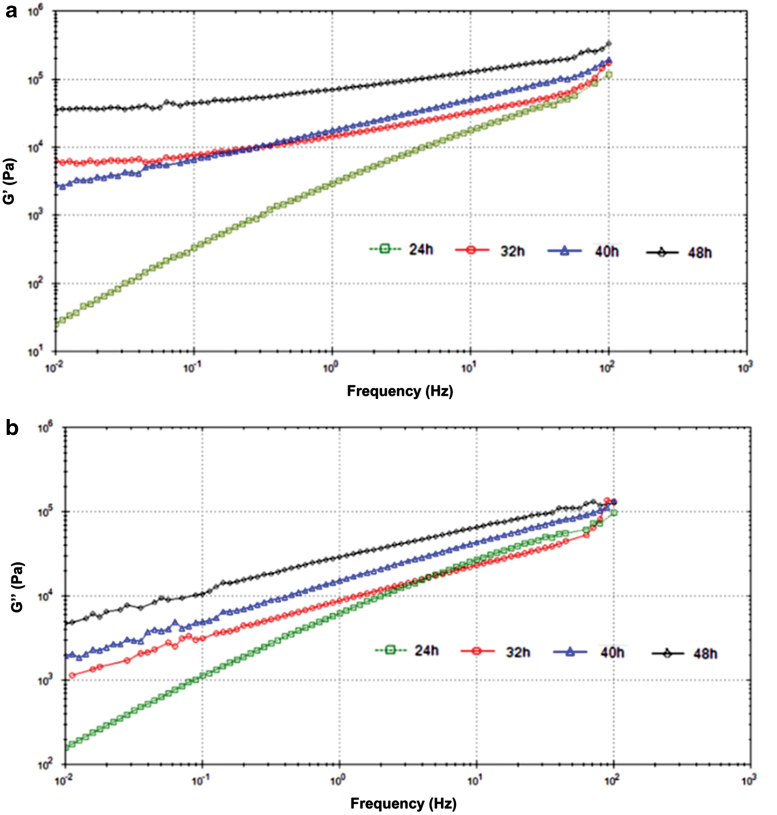

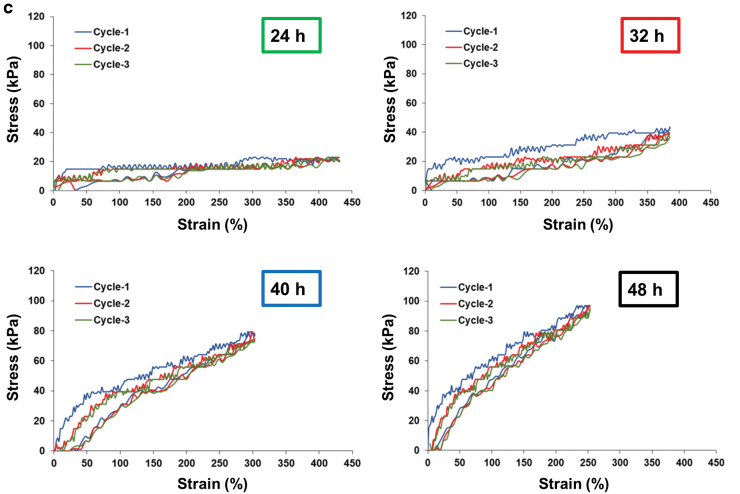
**(a)** Elastic (G′) and **(b)** viscous modulus (G′′) plots of PEGSA elastomers; **(c)** the cyclic loading graphs (stress vs. time). Color images are available online.

The elastic and viscous moduli were plotted against frequency (Fig. 7a, b). At 5 Hz, the elastic moduli were found as 10.64, 25.23, 36.85, and 105.2 kPa for the PEGSA elastomers cured for 24, 32, 40, and 48 h, respectively, while the viscous moduli were read as 17.75, 17.41, 31.96, and 51.15 kPa. As it can be seen from these values, the materials have dominantly viscous properties up to 24 h, however, they exhibited an increasing trend in the elastic modulus with time in parallel to the formation of the new crosslinks. According to the results of cyclic-load experiments (Fig. 7c; Supplementary Fig. S1), the elastomers did not exhibit hysteresis behavior between loading and unloading.

**FIG. 8.**
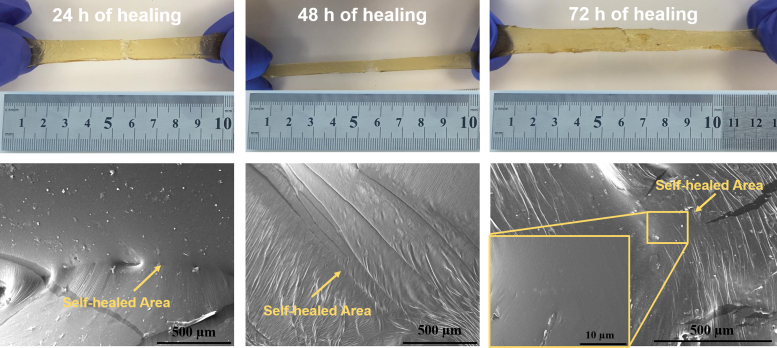
Self-healing properties of PEGSA elastomers after 24, 48, and 72 h of healing. Color images are available online.

Also, the self-healing capacity of PEGSA elastomer cured for 32 h was evaluated by cutting the polymer (3 × 1 cm) into two pieces and then letting them join together at room temperature for different times (Fig. 8). The obtained cut pieces were let to heal for 24, 48, and 72 h at room temperature, stretched following the same procedure to obtain the stress/strain curves and were examined, and SEM images were recorded. During the morphological analysis, it was observed that the join line was quite obvious in case of 24 h healed material, however, this line disappeared slowly as the healing time increased. This increase also contributed to the stretching capabilities of the material. SEM images taken after 24, 48, and 72 h of healing are in good agreement with the findings. At the end of 72 h, the self-healed area was observed to get better throughout the connected line (at 10 μm scale).

Elastomers healed for 24, 48, and 72 h were also analyzed mechanically; they were subjected to tensile test, following the previous procedure. The effects of healing period on mechanical features, tensile strength (kPa), elongation (%), and Young's modulus are provided in Table 2. The tensile strength and elongation of the self-healed elastomer (cured for 24 h) were found as 52 ± 10 kPa and 240.7% ± 20.2%, respectively. These values confirmed recovery of ∼50% of the strength and 34% of elongation values of the pristine material. As the healing period increased, tensile strength and elongation % increased as well. PEGSA elastomer healed for 72 h displayed 150% more elongation compared with elastomer healed for 24 h.

**Table 2.** Time-Dependent Recoveries of Mechanical Properties of Poly(Ethyleneglycol-sebacate) Elastomer Cut and Healed for 24, 48, and 72 h

Time points (h)	Tensile strength (MPa)	Elongation @ break (%)	Young's modulus (MPa)
24	0.052 (± 0.001)	240.7 (± 20.2)	0.0227 (± 0.002)
48	0.052 (± 0.010)	230.3 (± 65.4)	0.0250 (± 0.004)
72	0.062 (± 0.001)	391.3 (± 58.5)	0.0267 (± 0.001)

**FIG. 9.**
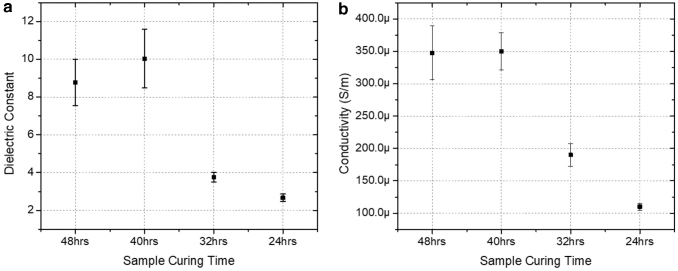
**(a)** Dielectric constant and **(b)** conductivity graphs of the samples cured for 24, 32, 40, and 48 h.

The electrical properties of the prepared elastomers were also investigated. Due to their actuation similar to muscles, dielectric elastomers have been subject to extensive research to extend their use in soft sensors, actuators, robotics, and so on.^20–22^ Therefore, particular emphasis has been given to the dielectric constant of the elastomer material. The high dielectric constant of the material indicates that it is suitable to be used as the medium of a capacitor. Materials with good flexibility and high dielectric constant are ideal for use as a capacitive sensor because the thickness of the medium changes easily for highly flexible materials. For capacitive sensors, it is critical to use a material with good dielectric constant to ensure reliable measurement of the capacitance. Figure 9a shows the dielectric constant (relative permittivity − $\varepsilon_{r}$) of PEGSA elastomer cured for 24, 32, 40, and 48 h. The curing time has a remarkable effect on the dielectric constant and so on the capacitance. Accordingly, PEGSA samples cured for higher time periods exhibited higher dielectric constants, whereas the greatest value was obtained for the PEGSA cured for 40 h. As the curing time increased, the dielectric constant showed an increase up until 40 h and then it droped, however, was still higher than the samples cured for lower times. As demonstrated in Figure 9a, the dielectric constant of PEGSA cured for 40 h ($\varepsilon_{r}=10$) outperforms many commercial elastomeric materials, including silicone (Nusil, CF19-2186, $\varepsilon_{r}=2.8$), acrylic (3M VHB 4910, $\varepsilon_{r}=4.8$), and IPN (VHB 4905-TMPTMA, $\varepsilon_{r}=2.43$).^23^ In addition, the capacitance measurements showed comparable values with some graphene-based elastomer composites.^24^

On the contrary, the conductivity increased with the increasing curing time of PEGSA (Fig. 9b). The mean conductivity of the elastomers cured for 40 h showed the greatest value and slightly higher than that cured for 48 h. The slight difference between the elastomers cured for 48 and 40 h may indicate that after a certain time of curing, the conductivity is not subject to significant change. The lowest conductivity was observed at the sample cured for 24 h.

**FIG. 10.**
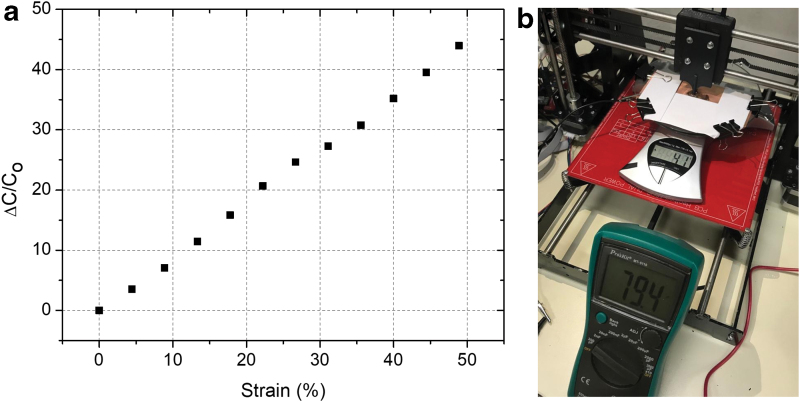
**(a)** Plot showing the change of capacitance as a function of strain (GF = 0.91). **(b)** A photograph of capacitive pressure sensing setup. GF, gauge factor. Color images are available online.

One of the indications for a good capacitive sensor is demonstrating the change of capacitance as a function of strain. According to Figure 10a, the increase in the uniaxial strain induced a linear increase in the capacitance change. The gauge factor of the material calculated from the fitting was around 0.91, whereas the theoretical limit is 1. The experimental results point out the promising use of this material for capacitive sensors. A representative photograph of the setup used for sensing applications is given in Figure 10b. In addition, to exemplify the use of the material for sensing applications, the video demonstrating its use as a pressure sensor is given as a Supplementary Video S1.

## Conclusion

In this study, we introduced an elastomer material that can be used in wearable sensors and soft robotics. This novel material can be defined as an elastomeric, self-healable, stretchable, transparent, and conductive polymer that is resulted from the chain reaction between diglycidyl ether groups and SA in the presence of caffeine as the catalyst followed by microwave irradiation, which created the crosslinked network. The FTIR and ^1^H NMR characterizations of the synthesized material showed that the esterification reaction took place between the polymer chains. The self-healing features are considered to result from the hydrogen bonds within the structure. Studies reported that weak bonds cannot provide sufficient mechanical features after recovery.^25,26^ Elastomer synthesized by Li *et al.* containing metal/ligand bonds had autonomous self-healing features at room temperature.^8^

The PEGSA elastomer produced in this study can be a promising candidate in the field considering its autonomous self-healing characteristics without needing the inclusion of metals or any further application of heat. The synthesized elastomer showed to mimic the same mechanical characteristics from the beginning more than 50% after 72 h of healing without needing any treatment. The stretching capability of the elastomer that can reach up to 400% after healing makes PEGSA superior compared with that of the currently reported self-healable elastomer.^14^ Besides self-healing and stretching capabilities, conductive features of the synthesized elastomer make it an ideal material for the capacitive sensors. There is no direct correlation between conductivity and dielectric constant of the samples. When compared with specific elastomers, the proposed elastomer of this study has the potential to be used as a resistive sensor.^27,28^ The flexibility of these materials favors their use as a resistive sensor, however, due to their relatively high dielectric constant, the elastomer prepared herein is also ideal to be used as a capacitive sensor.

## Supplementary Material

Supplemental data

Supplemental data
