# Supplementary material for: Microwave-Assisted Synthesis of Stretchable and Transparent Poly(Ethyleneglycol-Sebacate) Elastomers with Autonomous Self-Healing and Capacitive Properties
Source: Soft Robot. 2021 Jun 16;8(3):262–72. doi: 10.1089/soro.2019.0148 (PMC8236389; doi:10.1089/soro.2019.0148)
Supplement: Supplemental data [file Supp_FigS1.pdf]

Supplementary Data

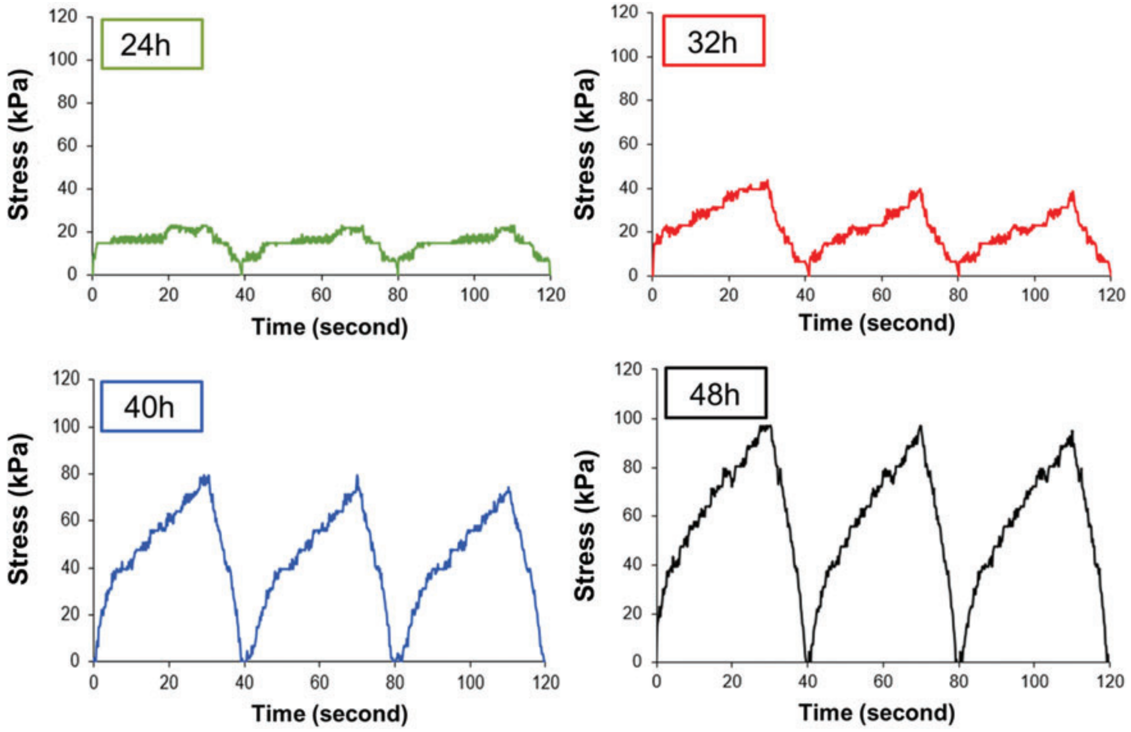

SUPPLEMENTARY FIG. S1. The cyclic loading graphs (stress vs. time) of PEGSA elastomers. PEGSA, poly (ethyleneglycol-sebacate).

SUPPLEMENTARY VIDEO S1. Autonomous capacitive sensing under applied pressure.
